# Supplementary material for: Immune responses to Mycobacterium tuberculosis membrane-associated antigens including alpha crystallin can potentially discriminate between latent infection and active tuberculosis disease
Source: PLoS One. 2020 Jan 31;15(1):e0228359. doi: 10.1371/journal.pone.0228359 (PMC6994005; doi:10.1371/journal.pone.0228359)
Supplement: S1 Text — (PDF) [file pone.0228359.s001.pdf]

S1 Text: Purchased Materials

Mycobacterium tuberculosis H37Ra (Mtb) was purchased from ATCC, USA (Cat No. 25177). PPD was purchased from Arkray Healthcare Pvt. Ltd, India. Peroxidase conjugated, affinity purified antibodies to human IgG (whole molecule), mouse IgG (whole molecule), human IgM (μ-chain specific) and human IgA (α-chain specific) were purchased from Sigma-Aldrich, USA. Fluorescent-tagged antibodies- anti-CD3-APC (clone UCHT-1) and anti-Ki67 (clone 20Raj1) were purchased from BD, USA and Thermo eBiosciences, USA respectively. Novex IgG subclass ELISA kit was purchased from Thermo, USA. Culture media and plastic ware were purchased from BD or Thermo, USA. All other chemicals were purchased from Sigma-Aldrich, USA.
