# Supplementary material for: Immune responses to Mycobacterium tuberculosis membrane-associated antigens including alpha crystallin can potentially discriminate between latent infection and active tuberculosis disease
Source: PLoS One. 2020 Jan 31;15(1):e0228359. doi: 10.1371/journal.pone.0228359 (PMC6994005; doi:10.1371/journal.pone.0228359)
Supplement: S1 Fig — (PDF) [file pone.0228359.s002.pdf]

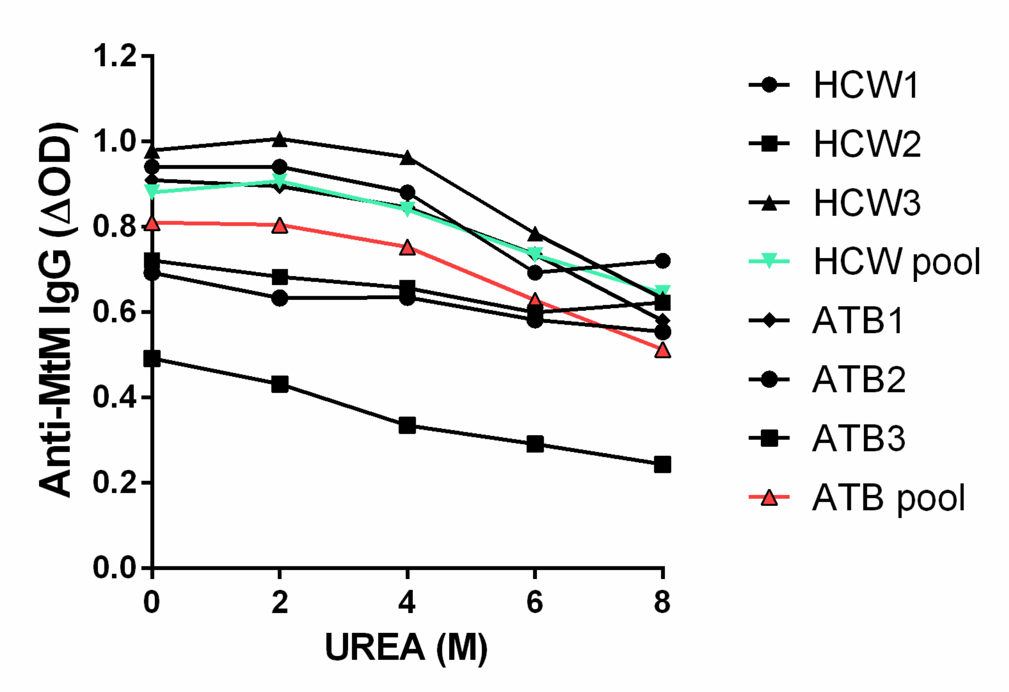

S2 Fig. Avidity determination for anti-MtM IgG antibodies by treatment with increasing concentrations of urea (0-8 M). IC50 value (urea concentration causing 50% inhibition of ΔOD) was not attained in 5 out of 6 test sera (HCW and ATB, individual and pooled).
