# Supplementary material for: Immune responses to Mycobacterium tuberculosis membrane-associated antigens including alpha crystallin can potentially discriminate between latent infection and active tuberculosis disease
Source: PLoS One. 2020 Jan 31;15(1):e0228359. doi: 10.1371/journal.pone.0228359 (PMC6994005; doi:10.1371/journal.pone.0228359)
Supplement: S2 Fig — (PDF) [file pone.0228359.s003.pdf]

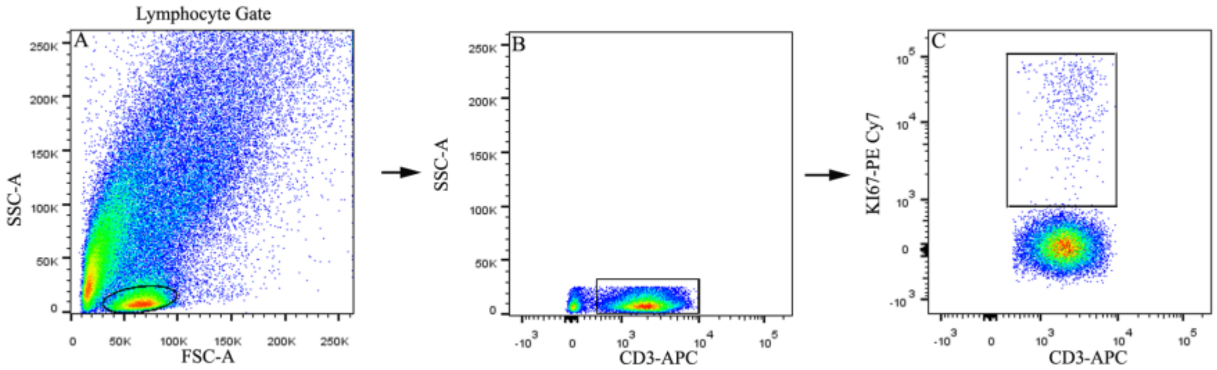

S3 Fig. Gating strategy. After the lymphocyte gate [A], CD3 gating was done [B]. Among the CD3+ cells, Ki67+ population was counted [C].
