# Supplementary material for: Immune responses to Mycobacterium tuberculosis membrane-associated antigens including alpha crystallin can potentially discriminate between latent infection and active tuberculosis disease
Source: PLoS One. 2020 Jan 31;15(1):e0228359. doi: 10.1371/journal.pone.0228359 (PMC6994005; doi:10.1371/journal.pone.0228359)
Supplement: S3 Fig — (PDF) [file pone.0228359.s004.pdf]

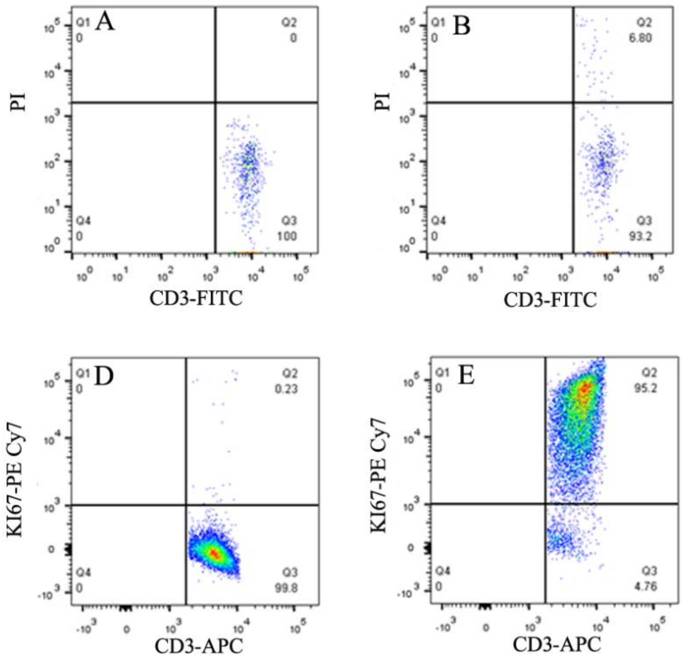

S4 Fig. Representative data on viability and proliferation capacity of T cells from a smear-positive TB patient. Cell viability, as determined by staining with propidium iodide, was 100% on day 0 (panel A) and >90% on day 6 (panel B). Panel C shows data for the positive control (cells treated with Triton X-100) in which all cells were dead. Day-6 data for cell-proliferative responses (% Ki67+ T cells) to the medium (panel D) and PHA (panel E) show that, in this case, 95% of CD3+ cells responded to the mitogen.
