# Supplementary material for: Immune responses to Mycobacterium tuberculosis membrane-associated antigens including alpha crystallin can potentially discriminate between latent infection and active tuberculosis disease
Source: PLoS One. 2020 Jan 31;15(1):e0228359. doi: 10.1371/journal.pone.0228359 (PMC6994005; doi:10.1371/journal.pone.0228359)
Supplement: S4 Fig — (PDF) [file pone.0228359.s005.pdf]

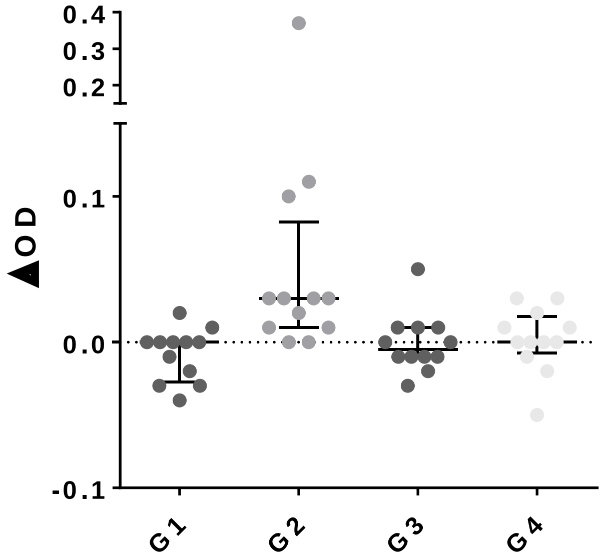

S6 Fig. Levels of IgG subtypes (G1-G4) against MtM in HCW sera. Columns show individual ΔOD values along with median and IQR. Corresponding P values are given in the Results section.
