# Supplementary material for: Immune responses to Mycobacterium tuberculosis membrane-associated antigens including alpha crystallin can potentially discriminate between latent infection and active tuberculosis disease
Source: PLoS One. 2020 Jan 31;15(1):e0228359. doi: 10.1371/journal.pone.0228359 (PMC6994005; doi:10.1371/journal.pone.0228359)
Supplement: S5 Fig — (PDF) [file pone.0228359.s006.pdf]

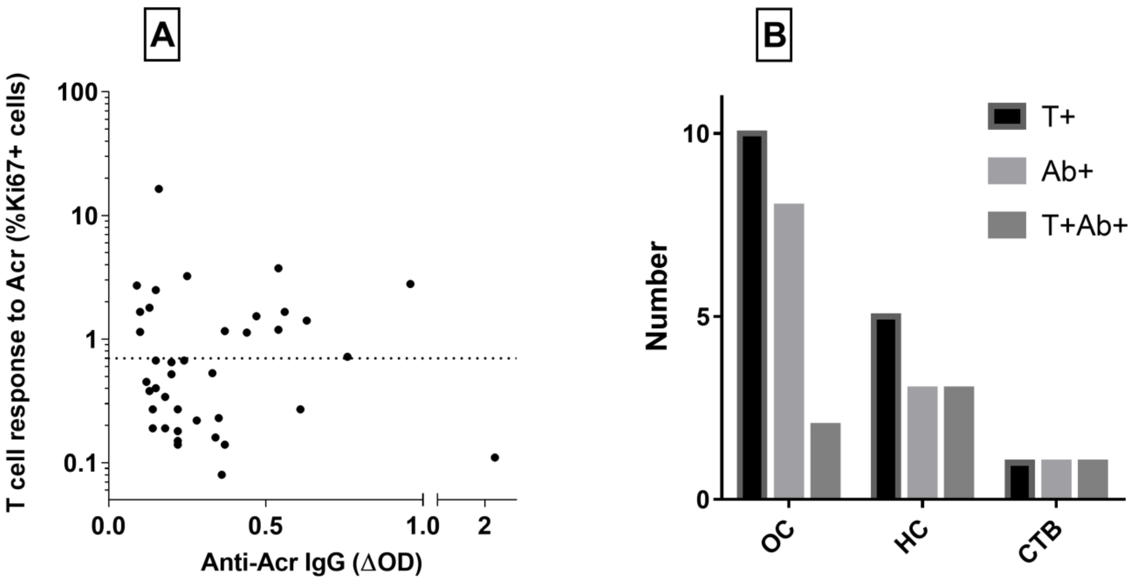

S7 Fig. Concordance between T cell and antibody responses to Acr. Panel A shows lack of correlation between T cell (dotted line denotes cut-off for positive T cell response) and antibody responses. Panel B shows concordance between positive T cell and high 'proportional' antibody ($\Delta OD_{Acr}/\Delta OD_{MtM}$ = >1) responses in the HCW categories OC, HC and CTB (T+, positive for T cell response; Ab+ positive for antibody response; T+Ab+, positive for both responses).
