# Supplementary material for: Immune responses to Mycobacterium tuberculosis membrane-associated antigens including alpha crystallin can potentially discriminate between latent infection and active tuberculosis disease
Source: PLoS One. 2020 Jan 31;15(1):e0228359. doi: 10.1371/journal.pone.0228359 (PMC6994005; doi:10.1371/journal.pone.0228359)
Supplement: S1 File — (PDF) [file pone.0228359.s007.pdf]

S8 File

Data for Fig 1 and Table 1

	Fig 1A			Fig 1B		
	MtM [IgG]	MtM [IgA]	MtM [IgM]	Acr [IgG]	Acr [IgA]	Acr [IgM]
OC						
	0.54	0.07	0.21	0.34	0.01	0.20
	0.43	0.03	0.07	2.16	0.02	-0.06
	0.58	0.09	0.03	0.22	0.06	-0.08
	0.29	0.03	-0.03	0.22	0.05	-0.16
	0.25	0.14	0.02	0.15	0.15	-0.16
	0.32	0.02	0.04	0.22	0.03	-0.04
	0.56	0.04	0.02	0.19	0.02	-0.06
	0.17	0.04	0.03	0.47	0.02	-0.19
	1.69	0.17	0.03	0.13	0.03	-0.05
	0.93	0.04	0.08	0.18	0.04	-0.07
	0.10	0.04	0.16	0.37	0.02	-0.10
	1.14	0.02	0.33	0.56	0.02	-0.21
	0.49	0.09	0.17	0.28	0.04	-0.08
	0.19	0.13	0.16	0.61	0.04	-0.19
	0.36	0.07	0.06	0.37	0.02	-0.03
	0.35	0.06	-0.01	0.32	0.05	-0.10
	0.18	0.04	0.04	0.12	0.06	-0.05
	0.18	0.03	0.10	0.13	0.03	-0.05
	0.20	0.04	0.02	0.13	0.04	-0.07
	1.96	0.08	0.03	0.96	0.13	-0.10
	0.10	0.02	0.37	0.10	0.03	-0.24
	0.17	0.06	0.03	0.33	0.03	-0.03
	0.27	0.06	0.05	0.24	0.01	-0.11
	0.12	-0.01	0.20	0.36	0.02	-0.14
	0.23	-0.03	0.17	0.14	0.02	-0.12
	0.31	0.06	0.08	0.76	0.05	-0.14
	0.92	1.10	0.07	0.25	0.06	-0.04
	0.26	0.18	-0.03	0.17	0.03	-0.04
	0.53	0.02	0.05	0.44	0.06	-0.10
	0.10	0.03	0.21	0.15	-0.01	-0.17
HC						
	0.49	0.04	0.20	0.35	0.00	-0.11
	1.75	0.02	0.06	0.14	0.02	-0.08
	0.38	0.07	0.38	0.54	0.03	-0.26
	0.33	0.03	0.18	0.54	0.01	-0.15
	0.37	0.19	0.01	0.10	0.02	-0.05
	0.23	0.06	0.19	0.15	0.03	-0.10
	0.06	0.04	0.06	0.09	0.03	-0.08
	0.30	0.03	0.03	0.16	0.06	-0.09

CTB						
	0.22	0.02	0.15	0.22	0.03	-0.07
	0.29	0.25	0.02	0.20	0.06	-0.04
	0.18	-0.02	0.17	0.18	0.01	-0.04
	0.27	0.07	0.18	0.63	0.02	-0.11
	0.66	0.06	0.04	0.20	0.03	-0.06
ATB						
	0.24	0.15	0.01	0.00	0.10	-0.01
	0.65	0.13	0.90	0.04	0.07	-0.05
	0.14	0.11	-0.06	0.39	0.05	-0.12
	0.05	0.03	-0.05	0.00	0.08	-0.13
	0.20	0.10	-0.02	0.06	0.06	-0.09
	1.64	0.45	0.00	0.70	0.17	-0.01
	0.29	0.06	0.02	0.21	0.04	-0.06
	0.92	0.01	-0.03	0.09	0.04	-0.09
	0.28	0.03	0.03	0.20	0.04	0.01
	0.05	0.14	-0.04	0.05	0.04	-0.07

Data for Fig 2A

OC-MtM (M)	HC-MtM (M)	CTB-MtM (M)	ATB-MtM (M)
0.21	0.20	0.15	0.01
0.07	0.06	0.02	0.90
0.03	0.38	0.17	-0.06
-0.03	0.18	0.18	-0.05
0.02	0.01	0.04	-0.02
0.04	0.19		0.00
0.02	0.06		0.02
0.03	0.03		-0.03
0.03			0.03
0.08			-0.04
0.16			
0.33			
0.17			
0.16			
0.06			
-0.01			
0.04			
0.10			
0.02			
0.03			
0.37			
0.03			
0.05			
0.20			
0.17			
0.08			
0.07			
-0.03			
0.05			
0.21			

Data for Fig 2B

OC-Acr (A)	HC-Acr (A)	CTB-Acr (A)	ATB-Acr (A)
0.01	0.00	0.03	0.10
0.02	0.02	0.06	0.07
0.06	0.03	0.01	0.05
0.05	0.01	0.02	0.08
0.15	0.02	0.03	0.06
0.03	0.03		0.17
0.02	0.03		0.04
0.02	0.06		0.04
0.03			0.04
0.04			0.04
0.02			
0.02			
0.04			
0.04			
0.02			
0.05			
0.06			
0.03			
0.04			
0.13			
0.03			
0.03			
0.01			
0.02			
0.02			
0.05			
0.06			
0.03			
0.06			
-0.01			

Data for Fig 3

MtM [G]		MtM [A]		MtM [M]		Acr [G]		Acr [A]		Acr [M]	
Buffer	Antigen	Buffer	Antigen	Buffer	Antigen	Buffer	Antigen	Buffer	Antigen	Buffer	Antigen
0.18	0.76	0.18	0.27	0.21	0.24	0.17	0.38	0.15	0.21	0.37	0.29
0.13	0.43	0.12	0.15	0.27	0.24	0.16	0.37	0.09	0.14	0.42	0.26
0.11	0.36	0.16	0.29	0.33	0.35	0.14	0.29	0.07	0.15	0.65	0.49
0.26	0.57	0.14	0.16	0.09	0.13	0.22	0.44	0.07	0.10	0.17	0.13
0.18	0.75	0.11	0.15	0.13	0.15	0.22	0.42	0.07	0.09	0.24	0.18
0.12	1.81	0.12	0.17	0.11	0.14	0.15	0.28	0.06	0.10	0.21	0.16
0.17	1.10	0.12	0.16	0.15	0.24	0.24	0.42	0.07	0.11	0.34	0.27
0.19	0.54	0.16	0.22	0.31	0.29	0.17	0.49	0.09	0.14	0.41	0.31
0.10	0.28	0.15	0.19	0.13	0.17	0.12	0.24	0.13	0.20	0.19	0.14
0.14	0.31	0.11	0.14	0.19	0.29	0.16	0.29	0.11	0.14	0.19	0.15
0.15	0.36	0.07	0.11	0.14	0.17	0.19	0.32	0.06	0.10	0.27	0.21
0.45	2.40	0.16	0.24	0.10	0.12	0.41	1.37	0.09	0.23	0.34	0.24
0.25	0.42	0.11	0.17	0.10	0.13	0.37	0.70	0.06	0.09	0.16	0.13
0.36	1.20	0.06	0.09	0.16	0.19	0.50	1.92	0.07	0.12	0.31	0.22
0.14	0.41	0.10	0.28	0.17	0.14	0.17	0.34	0.10	0.13	0.29	0.25
0.21	0.73	0.11	0.14	0.17	0.21	0.26	0.70	0.06	0.12	0.40	0.30
0.11	0.48	0.09	0.28	0.11	0.12	0.14	0.25	0.09	0.11	0.22	0.16
0.08	0.15	0.08	0.12	0.11	0.17	0.10	0.19	0.07	0.10	0.43	0.36
0.16	0.45	0.14	0.39	0.14	0.16	0.18	0.38	0.11	0.18	0.18	0.15
0.14	0.80	0.07	0.13	0.11	0.15	0.16	0.36	0.06	0.09	0.22	0.16

Data for Fig 4A

MtM IgG	Acr IgG
0.54	0.34
0.43	2.16
0.58	0.22
0.29	0.22
0.25	0.15
0.32	0.22
0.56	0.19
0.17	0.47
1.69	0.13
0.93	0.18
0.1	0.37
1.14	0.56
0.49	0.28
0.19	0.61
0.36	0.37
0.35	0.32
0.18	0.12
0.18	0.13
0.2	0.13
1.96	0.96
0.1	0.1
0.17	0.33
0.27	0.24
0.12	0.36
0.23	0.14
0.31	0.76
0.92	0.25
0.26	0.17
0.53	0.44
0.1	0.15
0.49	0.35
1.75	0.14
0.38	0.54
0.33	0.54
0.37	0.1
0.23	0.15
0.06	0.09
0.3	0.16
0.22	0.22
0.29	0.2

0.18	0.18
0.27	0.63
0.66	0.2
0.24	0
0.65	0.04
0.05	0
0.2	0.06
1.64	0.7
0.29	0.21
0.92	0.09
0.28	0.2
0.05	0.05
0.14	0.39

Data For Fig 4B

	MtM-G	Acr-G	Acr/MtM Ratio
OC			
	0.54	0.34	0.63
	0.43	2.16	5.02
	0.58	0.22	0.38
	0.29	0.22	0.76
	0.25	0.15	0.6
	0.32	0.22	0.69
	0.56	0.19	0.34
	0.17	0.47	2.76
	1.69	0.13	0.08
	0.93	0.18	0.18
	0.1	0.37	3.7
	1.14	0.56	0.49
	0.49	0.28	0.57
	0.19	0.61	3.21
	0.36	0.37	1.03
	0.35	0.32	0.91
	0.18	0.12	0.67
	0.18	0.13	0.72
	0.2	0.13	0.65
	1.96	0.96	0.49
	0.1	0.1	1
	0.17	0.33	1.94
	0.27	0.24	0.89
	0.12	0.36	3
	0.23	0.14	0.61
	0.31	0.76	2.45
	0.92	0.25	0.27
	0.26	0.17	0.65
	0.53	0.44	0.83
	0.1	0.15	1.5
HC			
	0.49	0.35	0.71
	1.75	0.14	0.08
	0.38	0.54	1.42
	0.33	0.54	1.64
	0.37	0.1	0.27
	0.23	0.15	0.65
	0.06	0.09	1.5
	0.3	0.16	0.53
CTB			
	0.22	0.22	1
	0.29	0.2	0.69
	0.18	0.18	1
	0.27	0.63	2.33

	0.66	0.2	0.3
ATB			
	0.24	0	0
	0.65	0.04	0.06
	0.14	0.39	2.79
	0.05	0	0
	0.2	0.06	0.3
	1.64	0.7	0.43
	0.29	0.21	0.72
	0.92	0.09	0.1
	0.28	0.2	0.71
	0.05	0.05	1

Data for Fig 5

OC			ATB		
Untreared	8M Urea	Avidity Index	Untreated	8M Urea	Avidity Index
1.07	0.59	55.14	0.27	0.06	22.2
1.24	0.77	62.1	0.13	0.01	7.69
0.91	0.56	61.54	0.45	0.20	44.4
1.04	0.56	53.85	0.97	0.38	39.1
1.78	1.77	99.45	1.50	1.06	70.67
0.58	0.33	56.9	0.95	0.53	55.79
0.56	0.23	41.07	0.50	0.15	30
0.40	0.20	50	0.42	0.06	14.29
0.69	0.28	40.58	0.88	0.46	52.27
0.50	0.18	36	1.26	0.95	75.4

Data for Fig 7
% Responder T cells
NA, not available

Medium	OC	HC	CTB	ATB
0.1	0.16	0.23	0.27	1.74
0.09	0.11	0.27	0.65	0.3
0.03	0.15	3.74	0.19	0.24
0.08	0.14	1.19	1.41	0.26
0.15	2.49	1.14	0.52	0.13
0.02	0.18	0.67		0.18
0.3	NA	2.71		0.1
0.11	1.53	16.4		0.54
0.14	1.79			0.47
0.31	0.34			0.24
0.19	0.14			
0.52	1.66			
0.09	0.22			
0.02	0.27			
0.35	1.16			
0.04	NA			
0.27	0.45			
0.03	NA			
0.84	0.38			
0.12	2.79			
0.57	1.66			
0.18	0.53			
0.67	0.67			
0.02	0.08			
0.3	0.19			
0.16	0.72			
0.19	3.23			
0.04	NA			
0.13	1.13			
0.15	0.4			
0.06				
0.04				
0.36				
0.31				
0.1				
0.22				
0.05				
0.03				
0.09				
0.18				
0.09				
0.06				
0.03				

0.17
0.18
0.15
0.13
0.06
0.13
0.06
0.17
0.24
0.03

Data for Fig 8A
[OD]

TST+	TST-	BCG+	BCG-
0.22	0.34	0.34	0.22
0.19	2.16	2.16	0.15
0.47	0.22	0.22	0.32
0.13	0.22	0.22	0.96
0.33	0.15	0.19	0.44
0.24	0.18	0.47	0.54
0.36	0.37	0.13	0.15
0.14	0.56	0.18	0.2
0.76	0.28	0.37	
0.25	0.61	0.56	
0.17	0.37	0.28	
0.44	0.32	0.61	
0.15	0.12	0.37	
0.14	0.13	0.12	
0.54	0.13	0.13	
0.54	0.96	0.13	
0.1	0.1	0.1	
0.15	0.35	0.33	
0.09	0.22	0.24	
0.16		0.36	
0.2		0.14	
0.18		0.76	
0.63		0.25	
0.2		0.17	
		0.15	
		0.35	
		0.14	
		0.54	
		0.1	
		0.09	
		0.16	
		0.22	
		0.18	
		0.63	
		0.2	

Data for Fig 8B
[OD]

TST+	TST-	BCG+	BCG-
0.32	0.54	0.54	0.58
0.56	0.43	0.43	0.25
0.17	0.58	0.29	0.35
1.69	0.29	0.32	1.96
0.17	0.25	0.56	0.53
0.27	0.93	0.17	0.33
0.12	0.10	1.69	0.23
0.23	1.14	0.93	0.29
0.31	0.49	0.10	
0.92	0.19	1.14	
0.26	0.36	0.49	
0.53	0.35	0.19	
0.10	0.18	0.36	
1.75	0.18	0.18	
0.38	0.20	0.18	
0.33	1.96	0.20	
0.37	0.10	0.10	
0.23	0.49	0.17	
0.06	0.22	0.27	
0.30		0.12	
0.29		0.23	
0.18		0.31	
0.27		0.92	
0.66		0.26	
		0.10	
		0.49	
		1.75	
		0.38	
		0.37	
		0.06	
		0.30	
		0.22	
		0.18	
		0.27	
		0.66	

Data For Fig 8C
% Responder T Cells
NA, not available

TST+	TST-	BCG+	BCG-
0.18	0.16	0.16	0.15
NA	0.11	0.11	2.49
1.53	0.15	0.14	NA
1.79	0.14	0.18	2.79
0.53	2.49	NA	1.13
0.67	0.34	1.53	1.19
0.08	0.14	1.79	0.67
0.19	1.66	0.34	0.65
0.72	0.22	0.14	
3.23	0.27	1.66	
NA	1.16	0.22	
1.13	NA	0.27	
0.4	0.45	1.16	
0.27	NA	0.45	
3.74	0.38	NA	
1.19	2.79	0.38	
1.14	1.66	1.66	
0.67	0.23	0.53	
2.71	0.27	0.67	
16.4		0.08	
0.65		0.19	
0.19		0.72	
1.41		3.23	
0.52		NA	
		0.4	
		0.23	
		0.27	
		3.74	
		1.14	
		2.71	
		16.4	
		0.27	
		0.19	
		1.41	
		0.52	

Data for Fig 9
% Responder T cells

MtM	Acr
0.85	0.16
1.95	0.11
0.83	0.15
0.79	0.14
3.31	2.49
1.73	0.18
19.2	1.53
3.94	1.79
1.99	0.34
1.18	0.14
9.84	1.66
0.85	0.22
1.4	0.27
7.34	1.16
3.17	0.45
1.29	0.38
3.83	2.79
13	1.66
1.26	0.53
5.02	0.67
0.6	0.08
3.58	0.19
5.7	0.72
5.06	3.23
2.78	1.13
0.69	0.4
3.16	0.23
12	0.27
13.4	3.74
0.59	1.19
1.39	1.14
5.92	0.67
0.93	2.71
17.6	16.4
0.87	0.27
1.34	0.65
1.18	0.19
2.62	1.41
3.78	0.52
