# Supplementary material for: Immune responses to Mycobacterium tuberculosis membrane-associated antigens including alpha crystallin can potentially discriminate between latent infection and active tuberculosis disease
Source: PLoS One. 2020 Jan 31;15(1):e0228359. doi: 10.1371/journal.pone.0228359 (PMC6994005; doi:10.1371/journal.pone.0228359)
Supplement: S1 Table — (PDF) [file pone.0228359.s008.pdf]

S5 Table. Significance of differences (P values) between levels of antibody isotypes (IgG, IgA and IgM) against the antigens MtM and Acr in study subjects (OC, HC, CTB and ATB). The significant differences ($P < 0.05$) are underscored.

Antibody Pairs	OC	HC	CTB	ATB
MtM IgG vs IgM	< 0.0001	0.0313	0.0625	0.0293
MtM IgG vs IgA	< 0.0001	0.0078	0.0625	0.0117
MtM IgA vs IgM	0.4261	0.2188	0.8125	0.1211
Acr IgG vs IgM	< 0.0001	0.0078	0.0625	0.002
Acr IgG vs IgA	< 0.0001	0.0078	0.0625	0.2031
Acr IgA vs IgM	< 0.0001	0.0078	0.0625	0.002
