# Supplementary material for: Immune responses to Mycobacterium tuberculosis membrane-associated antigens including alpha crystallin can potentially discriminate between latent infection and active tuberculosis disease
Source: PLoS One. 2020 Jan 31;15(1):e0228359. doi: 10.1371/journal.pone.0228359 (PMC6994005; doi:10.1371/journal.pone.0228359)
Supplement: Attachment — Submitted filename: Response to Comments on R1.docx [file pone.0228359.s010.docx]

**Review Comments to the Author**

**Reviewer #1:** The authors have undertaken substantial revision of the manuscript exploring humoral immunogenicity of membrane-associated proteins in human TB. The principle findings include (a) higher levels of IgM in contacts than in active TB patients (b) higher Acr IgA in active TB than contacts (c) altered ratios of IgG to Acr vs Mtm across the spectrum of TB disease.

In general, the comments has been addressed.

**Further comments:**

Comment-1. The major concern of statistical analysis has been resolved with the correction of non-parametric and multiple comparison tests.

(No response required)

C2. The rebuttal to the flow cytometry gating is accepted. The live/dead stain at day 6 confirms the cells were still alive in active TB.

(No response required)

C3. Thank you for the additional experiments regarding the antibody avidity. It is suggested to put the p-value in the figure given that the trend is discussed. Could the heterogeneity in antibody avidity in active TB be due to different durations of disease prior to presentation? Perhaps something to explore in future studies with greater powering.

Response: In the cited work [ref 5], authors had determined the avidity of anti-PPD antibodies in terms of molar concentration of urea required to reduce antibody bindings by 50% (IC50). Since they attained IC50 values in their experiment, statistical significance of differences could be calculated by *t*-test. However, in our case, the IC50 values were not attained (the possible reasons for which had been stated in revised manuscript R1). We nonetheless considered analysing the trends, but the data was insufficient. In any case, the two trends (HCW and ATB) appear comparable (S2 Fig). We had already stated in our previous revision (R1 Discussion) that the known heterogeneity in antibody responses of patients (which could be due to various reasons, including disease duration) and (perhaps more importantly) the differences in used protocol for avidity determinations could have caused these variations in avidity.

C4. The finding of IgM being greater in contacts than in active TB is interesting, suggesting the chronicity of active TB drives class-switching. Could a mechanism be included in the discussion?

Response: Thanks for pointing this out. We have modified the Discussion as suggested.

C5. The r-squared and p values should be inserted in the scatter plot figures (correlations).

Response: This has been done.

C6. The results regarding IgM not detectable against Acr is likely technical (concentration of Acr coating?), but the authors have addressed this clearly in the discussion. In the way it is presented, this could still be of benefit for the research community.

(No response required)

C7. Line 268: Can immunoglobulin levels truly be directly compared on an OD basis given it is being compared across different antigens and secondary antibodies? Would this not require a standard curve of subclasses to derive a specific concentration of immunoglobulin? However, it could be correct to state that IgG and then later for figure 6, IgG2 (line 316), is the most readily detectable immunoglobulins in their given assays.

Response: Thanks for pointing this out. The words ‘most abundant’ (line 268) and ‘dominant’ (line 316) have now been replaced by ‘most readily detectable’.

Minor comments:

1. Line 66: What is meant by ‘presumptive’ in single quotation marks?

R: It emphasizes the need for exercising caution while interpreting TST/IGRA results.

2. Line 88: Missing a word – Mtb membrane lysate or proteins?

R: Proteins (correction done).

3. Line 97: Why is ‘Mtb complex’ in single quotation marks?

R: Quotation marks have been removed.

4. Line 95-96: What is meant by sustain the bacilli in

R: Some of the dictionary meanings of sustain are: ‘to keep alive’, ‘strengthen’ or ‘support’.

5. Line 188: Should be IgA not AgA

R: Corrected.

6. Line 200: The concentration of the secondary should be included.

R: Included.

7. Line 256: Missing word – elaborated ‘on’.

R (line 276): Replaced the word elaborated with described.

8. Line 394: It is unclear what is meant by quantum of infection increasing.

R: We have replaced the word quantum with burden.

9. Line 556: Typo in semi-quantitative. The point with semi-quantitative is that levels of antibodies cannot be directly compared when using different reagents (secondaries) or assays. Further, it also poses a challenge for defining cut-off values for diagnostic tests as the units are arbitrary and contingent on the individual assay. However, it is accepted that this study is exploratory in nature.

R: Typo in line 566 has been corrected.

10. Line 451: Why is dynamic in single quotation marks?

R: Single quotation marks have been removed.

11. Line 492: Why is ‘cell-surface’ in single quotation marks?

R: To emphasize that ‘cell-surface’ is an ill-defined entity.

12. Line 512: “gets worsened” should perhaps be “is worsened”

R: Corrected.

**Reviewer #2:** My comments about some of the responses given by authors

Author’s response: We have used TST as the reference assay, reasons for which have now been elaborated more explicitly in revised Introduction (2nd paragraph).

Authors have explained the reason why they used TST as the reference assay. I may consider this a valid reason, but it would be easier to compare their results with others reports using IGRAs. Still the data provided in this paper may be useful to complement the information about this topic.

(No response required)

Author’s response: We fully agree with this observation. In fact we have not proposed that the crude membrane should be used as such. In concluding paragraph of Discussion we had said that “In addition to Acr, the study draws attention to MtM as an emerging source of immunodominant T cell antigens”. We have now revised the Discussion to further clarify this point. However, in real life the ‘non-tuberculous mycobacteria’ as well as BCG do not seem to confound the results of TST which also uses a crude antigen (Farhat et al, Int J Tuberc Lung Dis, 2006; 10:1192).

I agree with the paragraph mentioned in discussion. But the fact that MtM is a rich source of antigens, most of them unstudied, reveals that it is not close to determine which protein may actually function as biomarker. As author also responded: we have made some such attempts in the past (ref 8) but have not been able meet any remarkable success.

(No response required)

Additional comment:

I expected to see the changes made to the paper highlighted to facilitate the second review, instead I had to go through all the paper to see the changes

Response: As required by the journal, we had also submitted a copy of the manuscript showing corrections.
